# Offline Victimization, Psychological Morbidity, and Problematic Online Behavior among Chinese Secondary School Students

**DOI:** 10.3390/ijerph18189462

**Published:** 2021-09-08

**Authors:** Xiang Li, Daniel T. L. Shek, Esther Y. W. Shek

**Affiliations:** Department of Applied Social Sciences, The Hong Kong Polytechnic University, Hong Kong; daniel.shek@polyu.edu.hk (D.T.L.S.); esther.shek@polyu.edu.hk (E.Y.W.S.)

**Keywords:** victimization, depression, anxiety, Internet addiction, cyberbullying

## Abstract

Despite the rise of child victimization in different societies, few researchers have examined its consequences in terms of psychological morbidity (such as depression and anxiety) and problematic online behavior (such as Internet addiction and cyberbullying) in a single study. Moreover, no study has investigated the role of psychological morbidity in mediating the impact of victimization on problematic online behavior (indexed by Internet addiction and cyberbullying) in a single model. Based on a survey of 2843 Chinese secondary students (49.3% male; Mage = 13.97) from six public secondary schools in Fujian, China, we found that experience of victimization was positively associated with depression and anxiety, as well as Internet addiction and cyberbullying. Depression mediated the links between victimization and both Internet addiction and cyberbullying, with the mediating effect on Internet addiction found to be stronger for girls. While anxiety did not mediate the association between victimization and cyberbullying, it mediated the relationship between victimization and Internet addiction in boys. These findings enrich our understanding of the negative outcomes of victimization, as well as directions for intervention.

## 1. Introduction

Adolescence is a turbulent and transitional stage in which many young people experience many developmental challenges such as victimization. Hawker and Boulton [1] defined victimization as “the experience among children of being a target of the aggressive behavior of other children, who are not siblings and not necessarily age-mates (p. 441)”. Victimization is a multi-dimensional concept, and some scholars view physical victimization, verbal victimization, and relational victimization as the main forms of victimization e.g., [2,3]. Victimization is a negative developmental challenge with many short- and long-term negative consequences [4], which hinders children’s social adjustment and development [5]. Victimization has been identified as a tremendous threat to mental and social well-being [6,7]. As victimization is stressful, it creates heightened negative emotional reactions such as depression and anxiety [8,9]. Besides, being victimized may lead to problematic behaviors such as Internet addiction and cyberbullying [10,11].

### 1.1. Victimization and Psychological Distress

Victimization disrupts adolescents’ emotional and social development [12,13] and it results in psychological distress and maladjustment [14]. These include depression and anxiety, which are common indicators of psychological distress [15]: Dodge [16] showed that over 70% of adolescent victims suffered from depression due to their helplessness and hopelessness experiences; Craig [2] reported a higher level of anxiety amongst adolescents who had been victimized; Richard et al. [17] found that adolescents who had been victimized were very likely to be depressed and anxious; Stapinski et al. [18] revealed that both depression and anxiety were negative emotional outcomes of adolescent victimization. Hsieh et al. [10] further identified a positive relationship between victimization and different forms of psychological distress, including depression, anxiety, hostility, interpersonal sensitivity, and somatic symptoms. In short, peer victimization triggers depression and anxiety in adolescents [19,20]. However, the findings concerning gender differences in the impact of victimization on psychological distress are equivocal. While Stapinski et al. [18] showed that victimization had a similar effect on depression and anxiety for males and females, some studies suggest that there are gender differences involved: some studies showed that the related effect was stronger among girls than boys [21,22], whereas other researchers showed that the association was stronger in boys than girls [23].

### 1.2. Victimization and Adolescent Problematic Online Behavior

Online activity is an integral part of daily life, especially for adolescents, who make up the main body of Internet users. More than 97% of American young people and more than 85% of young people in China are Internet users [24,25]. As the Internet is a medium for breaking down barriers and connecting people who might be marginalized [26], it is a useful tool when adolescents cannot express themselves or get support in real life where they can express their needs and get help [27]. In other words, going online may help them escape adversities or solve difficulties because the Internet appears to be a safe place to avoid suffering. As such, adolescents who have been victimized may become addicted to the Internet where Internet addiction is characterized by “excessive or poorly controlled preoccupations, urges, or behaviors regarding computer use, and Internet access that leads to impairment or distress” [28] (p. 2). Some recent studies have reported a significant positive relationship between victimization and Internet addiction [10,21,29,30,31]. 

Besides Internet addiction, victimization may also breed cyberbullying behavior. As the Internet permits adolescents to say and do almost anything with anonymity and limited monitoring by adults [32], it can be an outlet for young people to seek revenge and resolve their victimization. Compared to traditional behaviors, the anonymity and invisibility of cyberbullying not only reduces the cost of aggression, but also provides online aggressors with a safer and more covert status. Thus, adolescents are more likely to be aggressive when they are online—this even can apply for those who are victims of offline bullying and are submissive and withdrawn in real life. As deviance can be an approach to alleviating or coping with stressful situations, real-life victims may seek revenge by assaulting the aggressor [33] or innocent individuals on the Internet as a way to address their anger and frustration [34]. In addition, being victimized will lead to cyberbullying via modeling (i.e., bullying others by observing the aggressor’s behavior). Empirically, some studies have revealed a positive link between victimization and cyberbullying in adolescents [12,35]. Moreover, Zsila et al. [36] reported that boys with experience of cybervictimization were more likely to engage in online bullying than girls. 

### 1.3. Mediating Effects of Psychological Distress on Problematic Online Behavior

In the eyes of some adolescents, seeking support over the Internet is a good way of responding to their victimization in real life, as it is an easy channel for building relationships with others [37]. According to Tsai et al. [38], victims with psychological distress in particular are more likely to develop psychological dependence on the Internet by spending more time online. For example, depression and psychological distress can intensify Internet addiction [10,39]. Li et al. [21] showed that both relational and physical victimization increased the depression and anxiety of adolescents, resulting in Internet addiction. They further showed that the mediating effect of depression and anxiety was stronger for females. Similarly, Hsieh et al. [40] demonstrated that psychological symptoms such as hostility, anxiety, and depression mediated the association between victimization and Internet addiction. 

Regarding depression as a mediator, Liang et al. [41] showed that boys were more likely to be addicted to the Internet when they felt depressed. Liu et al. [42] also showed that depression mediated the relationship between cybervictimization and Internet addiction. Furthermore, Jia et al. [29] reported that peer victimization increased the likelihood of adolescents’ Internet addiction by reducing their psychological security. Zhao et al. [31] also showed that the presence of meaning in life mediated the association between victimization and online game addiction among girls. Finally, Ha and Hwang [43] showed that female adolescents with psychological distress (e.g., depression) were more vulnerable to Internet addiction than did their male counterparts. There are also research findings showing that anxiety mediates the impact of victimization on Internet addiction. Weinstein et al. [28] suggested that individuals with a higher level of social anxiety are more likely to be addicted to the Internet. Feng et al. [44] found that anxiety or social anxiety contributed to Internet addiction because online communication relieved or eliminated negative emotions. 

Besides Internet addiction, people with psychological distress are more likely to engage in cyberbullying [34]. Adolescents with depression tend to transfer their negative emotions by carrying out cyberbullying [45]. In a meta-analysis of 81 empirical studies [46], depression was found to be a significant predictor of cyberbullying. Several research studies also revealed the positive relationship between depression and cyberbullying: Kırcaburun et al. [47] showed that depression predicted cyberbullying; Martínez–Monteagudo et al. [48] found that participants with higher levels of depression tended to display more cyberbullying behavior; Richard et al. [17] reported that mental health problems such as depression and anxiety mediated the association between victimization and some problem behaviors including drinking, smoking, gambling, and drug use. 

There are also studies showing the mediating role of psychological morbidity in the influence of victimization and cyberbullying: Luk et al. [22] showed that depression mediated the relationship between victimization and substance use for female adolescents in the United States; Cénat et al. [49] reported that psychological distress was a mediator between cybervictimization and later substance use; Kim et al. [50] showed that depression was a mediator between different types of victimization and alcohol use. However, no study has to date examined the mediating effect of depression in the link between victimization and cyberbullying. Similarly, very few studies have explored the association between anxiety and cyberbullying [51,52].

### 1.4. The Present Study

There are several research gaps in the literature on the consequences of victimization. First, research on the consequences of victimization in terms of psychological morbidity (such as depression and anxiety) and problematic online behavior (such as Internet addiction and cyberbullying) is still in its infancy. Hence, we need answers on: (a) the relationship between victimization and psychological morbidity; (b) the relationship between victimization and problematic online behavior; and (c) the relationship between psychological morbidity and problematic online behavior. Second, as few studies have examined the mediating role of psychological distress (depression and anxiety) on the influence of victimization on problematic online behavior, there is a need to conduct related studies to enrich the theory on the consequences of victimization. Third, it is noteworthy that there are very few studies covering different Asian societies, including China. Empirical research studies based on Chinese adolescents is important for three reasons. First, the huge number of Chinese adolescents clearly suggests that data from Chinese adolescents should be collected to examine generalizability of theoretical models in the field, which are mostly developed in a Western context. In fact, the occurrence of aggressive incidents in schools in China is increasing rapidly, with the estimated rate of victimization being 20% [53]. Second, regarding the possible inter-relationships amongst victimization, psychological morbidity, and problematic online behavior, few studies have used multiple indicators of psychological distress and problematic online behavior. In this paper, we argue that two indicators of psychological distress (depression and anxiety) should be used. Depression and anxiety should be examined because they are common indicators of mental health and victimization leads to depression and anxiety symptoms [18]. Besides, we argue that both Internet addiction and cyberbullying should be examined for problematic online behavior. While Internet addiction is non-aggressive, making it similar to internalizing behavior, cyberbullying is aggressive in nature, which is similar to externalizing behavior. Finally, this study is the first one to examine developmental pathways from victimization to problematic online behaviors by exploring the underlying mediating mechanisms of psychological morbidity based on the social information processing (SIP) model [54]. The SIP model maintains that an individual responds to a social situation (e.g., victimization) through sequential information-processing steps. It implies that the selection of different reactions (e.g., problematic online behavior) depends on a series of social information processing events, such as individual emotional states and emotional regulation skills [53,55]. 

Against this background, this study explored the psychological distress and problematic online behavior of adolescents after victimization in real life. It attempted to investigate the interplay between real life victimization and the cyberworld by exploring how adolescents’ online behaviors develop after victimization. In particular, we studied the mediating mechanisms of psychological distress (i.e., depression and anxiety) on the relationship between victimization and problematic online behaviors, including Internet addiction and cyberbullying. We attempted to answer the following four research questions in this study: 

Research Question 1: Does adolescent victimization lead to emotional distress? Based on the existing literature [10,18], it was expected that adolescents’ victimization experiences in real life would be positively associated with psychological distress indexed by depression (Hypothesis 1a) and anxiety (Hypothesis 1b). 

Research Question 2: What is the relationship between victimization in the real world and problematic online behavior? Based on the existing literature [11,29], it was expected that adolescents’ victimization experiences in real life would be positively associated with problematic online behavior indexed by Internet addiction (Hypothesis 2a) and cyberbullying (Hypothesis 2b). 

Research Question 3: What is the relationship between psychological distress and problematic online behavior? Based on the existing literature [10,21], it was expected that psychological distress indexed by depression would be positively related to Internet addiction (Hypothesis 3a) and cyberbullying (Hypothesis 3b). Besides, it was hypothesized that psychological distress indexed by anxiety would be positively related to Internet addiction (Hypothesis 3c) and cyberbullying (Hypothesis 3d). 

Research Question 4: What is the mediating role of psychological distress indexed by depression and anxiety in the link between victimization and problematic online behavior indexed by Internet addiction and cyberbullying? With reference to the existing literature [21], it was expected that adolescent depression would mediate the relationship between victimization and Internet addiction (Hypothesis 4a) as well as cyberbullying (Hypothesis 4b). Besides, it was proposed that anxiety would mediate the influence of victimization on Internet addiction (Hypothesis 4c) and cyberbullying (Hypothesis 4d).

## 2. Materials and Methods

### 2.1. Participants

A total of 2848 Grade 7 and Grade 8 students at six secondary schools in Fujian Province, China, participated in this study. All six schools are public schools, which are the most common type of schools in China. To avoid the problem of missing values, we removed five cases that did not report gender and did not report on more than three of the study variables. Of the 2843 students who completed the questionnaire in full, 1403 were male and 1440 were female. The students’ ages ranged from 12 to 17 years (Mage = 13.97, SD = 0.84). There was a relatively even spread of participants across the two grade levels (1421 Grade 7 students and 1422 Grade 8 students) included in the sample. 

### 2.2. Procedures 

Participation in this study was completely voluntary. Ethical approval from the institutional review board of the University, approval for data collection from the participating schools, parental permission, and the students’ own consent had been obtained before the data collection. The students completed a paper-and-pencil self-report questionnaire under the supervision of trained research assistants in their own classrooms. All information provided by the respondents was treated as strictly confidential. The students took around 15 minutes to complete the questionnaire.

### 2.3. Measurement 

#### 2.3.1. Victimization 

The Multidimensional Peer-Victimization Scale (MPVS) [56] was used to measure victimization in real life. The MPVS is a widely used measure globally with good psychometric properties [57]. Responses are collected using a three-point Likert scale (0 = not at all, 1 = once, and 2 = more than once) with 16 items (e.g., “Beat me up”). The MPVS measures different forms of victimization, including physical, verbal, and social victimization, and damage to property, and had a good reliability of 0.91 in this study. Higher scores suggest more victimization experience. This scale has been validated in China, including mainland China and Hong Kong [53]. 

#### 2.3.2. Depression 

A short form of the Center for Epidemiologic Studies Depression Scale (CES-D Scale) [58] with 10 items (e.g., “I felt depressed”) was used to measure depressive symptoms among nonclinical, subclinical, and clinical populations [59]. Responses are collected using a four-point Likert scale ranging from 0 = rarely or none of the time to 3 = most or all of the time. A higher score indicates a higher level of depression. The reliability of this scale in this study is good (0.79). This scale has been validated and widely used in mainland China [53,60]. 

#### 2.3.3. Anxiety 

The Hospital Anxiety and Depression Scale (HADS) is a 14-item self-assessment measure that detects states of depression and anxiety [61]. Its good psychometric properties and comprehensiveness make it commonly used as a screening measure for psychological morbidity in both clinical and nonclinical samples [62,63]. To meet the specific requirements of this research, we used the seven-item subscale to measure anxiety (e.g., “worrying thoughts go through my mind”). Responses are collected using a four-point Likert scale ranging from 0 = not at all to 3 = most of the time. A higher score indicates a higher level of anxiety. Scale reliability was good in this study, at 0.84. This scale has been validated in mainland China [64]. 

#### 2.3.4. Internet Addiction 

A short version of the Internet Addiction Test (IAT) [65,66] was used to assess Internet addiction in terms of loss of control, time management and craving, and social problems. The original IAT has been widely used in a large number of studies in different countries, while the short version of the IAT demonstrates a clearer factor structure and is easy to administer [65]. This 12-item instrument asks how personal Internet use has affected individuals’ daily routine, sleeping patterns, feelings, productivity, and social life (e.g., “How often do you find that you stay online longer than you intended?”). Responses are collected using a five-point Likert scale ranging from 1 = never to 5 = very often. A higher score suggests more serious Internet addiction. The reliability of the scale in this study was good, at 0.90. This scale has been conducted in mainland China [67]. 

#### 2.3.5. Cyberbullying 

The nine-item Cyberbullying Questionnaire [32] was used to measure the prevalence and frequency of cyberbullying and covers activities such as broadcasting, deception, and online actions targeted at the person (e.g., “I made fun of someone by sending/posting stories, jokes, or pictures about him/her”). It systematically assesses the nature and characteristics of cyberbullying and it has been widely used in different studies. Responses are collected using a five-point Likert scale ranging from 1 = never to 5 = about a few times every week. A higher score suggests a higher level of cyberbullying involvement. The reliability of this scale was good at 0.80 in this study. This scale has been used in Asian societies such as Singapore [32].

### 2.4. Data Analysis Plan 

We firstly conducted descriptive analyses of all the study variables, using SPSS 26.0 (IBM, Armonk, NY, USA). The correlations among the latent constructs of this study were then analyzed by Mplus 8.0 (Muthén & Muthén, Los Angeles, CA, USA). To investigate the mediating role of depression and anxiety on the links between victimization and each of Internet addiction and cyberbullying, we employed structural equation modeling. A two-step modeling approach [68] was adopted. The measurement model examines the relationships between the latent constructs and their observed indicators, while the structural model examines the intercorrelations among the latent constructs. A series of model fit indices, including the comparative fit index (CFI), the Tucker–Lewis Index (TLI), the root mean square error of approximation (RMSEA), and the standardized root mean squared error (SRMR), were used to evaluate the goodness of fit of the model. A CFI and TLI ≥ 0.95 are considered a superior fit; an RMSEA ≤ 0.06 and an SRMR ≤ 0.08 also indicate a good fit [69,70]. Bootstrapping was performed by resampling 1000 times with calculation of confidence intervals (CIs). This study examined the 95% CI. If there is a 95% CI for the indirect effect excluding the value of “zero”, this suggests statistically significant mediation. The proposed mediator would be deemed a complete mediator if the relationships between the predictor and the outcomes became statistically nonsignificant. In contrast, it would be deemed a partial mediator if these relationships remained statistically significantly but less so. Finally, to compare whether there were any statistically significant differences in the mediation hypotheses between males and females, multiple-group analyses with gender as the group variable were conducted and estimates compared using the Wald chi-square test of parameter equalities.

## 3. Results

The mean values, standardized deviations, and reliability estimates among victimization, depression, anxiety, Internet addiction, and cyberbullying are presented in Table 1.

### 3.1. Measurement Model

To reduce sampling errors and the likelihood of correlated residuals and dual loadings while considering other advantages of models based on parcels (e.g., lower likelihood of distributional violations, higher reliability), we created item parcels for depression, anxiety, and cyberbullying by balancing the fact loadings. The measurement model includes five latent constructs, including victimization with four parcels, depression with three parcels, anxiety with three parcels, Internet addiction with two parcels, and cyberbullying with three parcels. Additionally, we created four parcels for victimization (i.e., one each for physical, verbal, and social victimization, and damage to property) and two parcels for Internet addiction (i.e., one each for loss of control/time management and craving/social problems). The results of the measurement model revealed a good model fit: χ^2^ (80, *n* = 2843) = 761, *p* < 0.001, with a CFI of 0.967, TLI of 0.957, RMSEA of 0.055 (90% CI: 0.051–0.058), and SRMR of 0.028. The five latent constructs were significantly correlated with one another (see Table 2).

### 3.2. Structural Model

**Figure 1:**
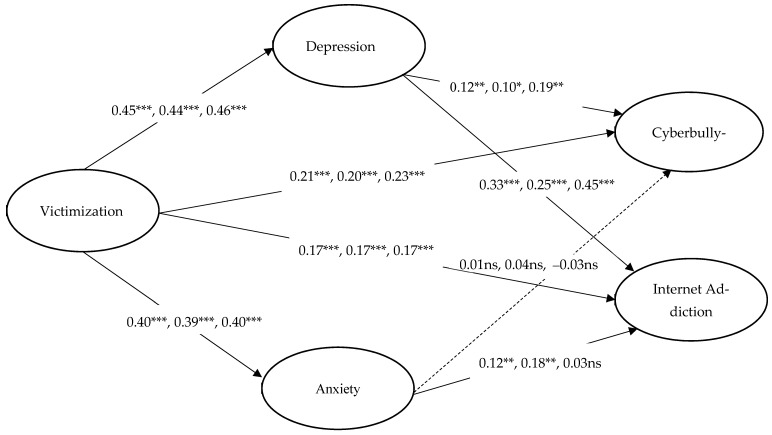
Structural equation model. Latent constructs are shown in ellipses. The standardized coefficients of all paths from left to right represent the entire, male, and female samples, respectively, *** *p* < 0.001; ** *p* < 0.01; * *p* = 0.05; ns = non-significant.

To investigate the proposed hypotheses, a structural model was examined, with gender and age treated as covariates to control for their effects on all constructs involved in the mediation model. The structural model (see Figure 1) fit the data well: χ2 (100, *n* = 2843) = 1121, *p* < 0.001, with a CFI of 0.952, TLI of 0.935, RMSEA of 0.060 (90% CI: 0.057–0.063), and SRMR of 0.032. The results showed that victimization was strongly related to depression (β = 0.45, *p* < 0.001) and anxiety (β = 0.40, *p* < 0.001), supporting Hypotheses 1a and 1b. We further found support for Hypotheses 2a and 2b where victimization strongly predicted Internet addiction (β = 0.17, *p* < 0.001) and cyberbullying (β = 0.21, *p* < 0.001). However, while depression significantly linked to both Internet addiction (β = 0.33, *p* < 0.001) and cyberbullying (β = 0.12, *p* < 0.01), anxiety only linked to Internet addiction (β = 0.12, *p* < 0.01) but not cyberbullying (β = 0.01, *p* > 0.05). Thus, only hypotheses 3a to 3c were supported. We found a significant indirect effect of depression in the association between victimization and Internet addiction (β = 0.15, *p* < 0.001, 95% CI = [0.11, 0.19]) and cyberbullying (β = 0.06, *p* < 0.01, 95% CI = [0.02, 0.09]) because zero was not included in the 95% CIs. These findings showed that depression served as the mediator of the relationships between victimization and both Internet addiction and cyberbullying. Thus, hypotheses 4a and 4b were confirmed. On the other hand, anxiety mediated the relationship between victimization and Internet addiction only. We found a significant indirect effect of anxiety in the association between victimization and Internet addiction (β = 0.05, *p* < 0.01, 95% CI = [0.02, 0.08]) because zero was not included in the 95% CIs, and a nonsignificant indirect effect of anxiety in the relationship between victimization and cyberbullying (β = 0.01, *p* > 0.05, 95% CI = [−0.03, 0.04]). Thus, only Hypothesis 4c was supported. After controlling for depression and anxiety, the direct effect of victimization on Internet addiction (β = 0.17, *p* < 0.001) and cyberbullying (β = 0.21, *p* < 0.001) was still statistically significant, indicating that depression and anxiety partially mediated the relationships between victimization and both Internet addiction and cyberbullying.

### 3.3. Gender Differences

To compare the differences in every path in the structural model across gender, the Wald chi-square test of parameter equalities was performed (see Table 3). Results showed that the structural path from victimization to depression was significantly different between males and females (Wald test value (1) = 7.16, *p* < 0.01), with a stronger predictive effect for girls (βmale = 0.44, *p* < 0.001; βfemale = 0.46, *p* < 0.001). This means that victimization experiences made girls more depressed than boys. Besides, the path from depression to Internet addiction was significantly different between males and females (Wald test value (1) = 4.50, *p* < 0.05), with a stronger predictive effect for girls (βmale = 0.25, *p* < 0.001; βfemale = 0.45, *p* < 0.001). This suggests that depression made girls more likely to be addicted to the Internet than boys. Additionally, the association between anxiety and Internet addiction was significantly different between males and females (Wald test value (1) = 5.87, *p* < 0.05), with a significant predictive effect for boys only (βmale = 0.18, *p* < 0.01; βfemale = 0.03, *p* > 0.05). This means that anxiety only made boys more likely to be addicted to the Internet. The mediation path test found that the path from victimization to Internet addiction via depression was significantly different between males and females (Wald test value (1) = 8.69, *p* < 0.01), with the mediating effect of depression on the association between victimization and Internet addiction found to be stronger for girls (βmale = 0.11, *p* < 0.001, 95% CI = [0.06, 0.16]; βfemale = 0.21, *p* < 0.001, 95% CI = [0.15, 0.27]). A gender difference was also found for the influence of victimization on Internet addiction via anxiety (Wald test value (1) = 4.40, *p* < 0.05) where anxiety was a significant mediator of the relationship between victimization and the Internet addiction link for boys but not for girls (βmale = 0.07, *p* < 0.001, 95% CI = [0.03, 0.11]; βfemale = 0.01, *p* > 0.05, 95% CI = [−0.03, 0.06]).

## 4. Discussion

As a pioneering study, this research provides an initial basis for exploring the relationships between real life experience of victimization and adolescent emotional responses and problematic online behavior. Drawing on a sample of approximately 3000 school-aged adolescents from six secondary schools in mainland China, we found a positive association between victimization experiences in real life and each of participants’ negative emotional responses (e.g., depression and anxiety) and problem behavior in the virtual world (e.g., Internet addiction and cyberbullying). Moreover, we found that depression resulting from victimization further increased the likelihood of being addicted to the Internet and engaging in cyberbullying, whereas anxiety prompted by victimization mediated its association with Internet addiction. 

There are only very few studies that have investigated the association between victimization, psychological distress, and problematic online behavior in one single study e.g., [10,21]. Regarding psychological distress, existing studies mainly focus on depression e.g., [23,39,71], with very few studies examining the role of anxiety in this area. As such, this study has enriched our understanding of the association between anxiety and problematic online behavior indexed by Internet addiction and cyberbullying. Regarding the consequences of psychological distress in the virtual world, previous studies mainly focus on Internet addiction, with very few studies examining cyberbullying [3,15]. In short, one unique feature of this study is examination of the association between offline victimization and online cyberbullying by adding the meditating effects of both depression and anxiety. 

In line with previous studies e.g., [18,72], we revealed that victimization experiences in real life significantly increased adolescents’ psychological distress, such as depression and anxiety, which supported Hypothesis 1a and Hypothesis 1b. This implies that victimization can have negative and serious psychological consequences for young people. After being bullied, adolescents usually feel helpless and hopeless, leading to depression [72]. Such victimization experiences could make adolescents anxious, resulting in feelings such as being scared and nervous towards being bullied again in the future. Consistent with some existing studies [21,22], we have also found that victimization resulted in a higher level of depression among females, suggesting that girls are more vulnerable than boys after being bullied. A possible explanation is that females care more about their interpersonal relationships and see being bullied as a failure in this domain [73]. Moreover, girls tended to blame themselves and adopt negative strategies to cope with distressing situations compared to boys [30]. This indicates that it is necessary to provide appropriate counselling services for adolescents who have experienced victimization in daily life. In particular, more attention should be paid to girls, who are prone to have more negative emotions. The findings suggest that the negative impact of victimization on psychological distress is rather universal across cultures. 

As expected, victimization in the real world was significantly and positively related to problematic online behaviors, including Internet addiction and cyberbullying (Hypothesis 2a and Hypothesis 2b). This implies that adolescents who have experienced victimization in real life are more likely to be addicted to the Internet and engage in cyberbullying. Besides, the findings supported Hypothesis 3a to Hypothesis 3d that psychological distress indexed by depression and anxiety was positively associated with Internet addiction and cyberbullying. As victimization reduces the level of psychological security, adolescents with lower security may have used their virtual life to counteract the resulting sense of emptiness [29]. Adolescents who have suffered victimization tend to escape from reality and engage in the virtual world because it seems to be a safe space for them to escape from unwanted suffering in real life [10], in particular when they do not know how to handle the negative consequences of being bullied [74,75]. Furthermore, socially disadvantaged groups (e.g., adolescent victims) may lack traditional forms of social support such as limited peer support, thus making them more inclined to communicate with, and seek help from, the Internet. Besides, consistent with the findings of Jang et al. [11], we also found that adolescents experiencing offline victimization tended to engage in cyberbullying. Due to the anonymity and ease of engagement of such activities, victims would feel more empowered to seek revenge against their bullies or imitate them in order to bully others online. First, people with victimization experiences in real life are more likely to bully others, given that bullying is a learnt behavior possibly via modeling [36]. Second, victims may want others to understand and experience their feelings of being bullied by bullying online [12]. Last but not least, cyberbullying is easier for adolescents to conduct because it neutralizes the disadvantage posed by a lack of physical power in real life [11,12].

Through exploring the mediation of the link between victimization and Internet addition, we found that victimization was related to an increase in depression and anxiety, which further increased Internet addiction (Hypotheses 4a and 4c). Several possible processes may be involved in such observations. First, when adolescents face negative situations (e.g., real-life victimization), they are more likely to seek out happiness and satisfaction via entertainment tools (e.g., WeChat, QQ) because these actions could reduce loneliness and depressive symptoms [74,76,77]. Second, the damage to self-worth and self-esteem caused by victimization would make individuals more afraid of the world in which they reside and retreat further to the online world [11], while increasing Internet use can alleviate anxiety caused by stress [44]. Third, loneliness and poor social skills in real life drive adolescents to engage in more online interaction [28,78] and avoid real life, face-to-face interaction [79]. When people are surfing the Internet, social anxiety can decrease because face-to-face communication is not required and they can hide themselves online [21]. As victimization may make young people anxious when going about their real life, they may be less comfortable communicating with people for fear of being victimized again; accordingly, immersing themselves in the online world should be a good way to relieve anxiety. 

Unlike the findings of Li et al. [21] that depression and anxiety have stronger mediation effects on the association between victimization and Internet addiction for girls, our study reveals that while the mediation effect of depression on this relationship is stronger for females, anxiety mediated this link for males but not for females. One possible reason for this finding is that in our study, depression was found to increase more Internet addictive behaviors in girls than in boys. Primarily, females with emotional difficulties are more likely to become addicted to the Internet because it can help to enhance social networking and interpersonal relationship through chatting online [43]. Besides, female students with victimization experiences reported low levels of presence of meaning, which in turn increased their addiction to online life [60]. Interestingly, we found that anxiety did not mediate the association between victimization and Internet addiction, and indeed had no relationship with Internet addiction for females. Although victimization would make both male and female feel anxious, anxiety results in Internet addiction for boys only and not girls. This implies that online interaction and virtual life may only help males to reduce their anxiety while females may use different coping strategies. 

Our study further highlights the mediators in the relationship between victimization and cyberbullying. Depression was found to mediate the relationship between offline victimization and cyberbullying (Hypothesis 4b) where poor psychological health is associated with cyberbullying [12]. Hsieh [80] pointed out that adolescents are more likely to display cyberbullying when under parental psychological control because their revenge motivation will increase under these circumstances. Revenge activities such as cyberbullying may help stressed adolescents to reduce their negative emotions; it may also make them feel more powerful and dominant. It may be noted that although anxiety is another common emotional response in adolescents with experience of victimization, it did not mediate the relationship between offline victimization and cyberbullying (Hypothesis 4d). Such a finding is consistent with previous work suggesting that there is no causal relationship between anxiety and cyberbullying (e.g., [52]). This may be due to the possibility that people with anxiety normally feel scared and nervous towards communicating with others, and so are less likely to cyberbully people. Due to the very limited research on the link between negative emotions and cyberbullying, this mechanism deserves further study.

While our findings expand the theoretical account of the association between offline victimization and problem behavior in the virtual world, as well as the underlying mediating mechanisms of negative emotional responses (i.e., depression and anxiety), three limitations should be acknowledged. First, although the sample size in the current study was large, all respondents were from Fujian Province. The findings cannot be generalized to all Chinese adolescents given that there are 23 provinces, five autonomous regions, four municipalities, and two special administrative regions in China. The findings of this study need to be replicated in other areas of China as well as other parts of the world in the future. Second, as all the measures used in the current study were self-reported by adolescents themselves, the possibility of common method variance should be noted. As the respondents may not have accurate recall of the related behavior, multiple informants should be invited to provide more accurate and unbiased responses. Last but not least, the current study was a cross-sectional design, which precludes any conclusions about the causality and directionality of these relationships over time. A longitudinal study would contribute to our understanding of the relationships tested in the current study. 

## 5. Conclusions

The rapid development of Internet technology has shortened the distance between cyberspace and real life, making the boundary between online and offline life less clear. This is especially true for adolescents, who are its main users. This study has enriched our understanding on the impact of real-life victimization experiences on adolescents’ emotional responses as well as their typical problematic online behaviors. Undoubtedly, gathering more knowledge about the outcomes of victimization in real life and the mechanisms underlying the links between such experiences and problematic online behavior can help us design more effective prevention and intervention programs to help adolescents who have been victimized to avoid conducting their own problem behaviors online. Specifically, depression and anxiety as potential consequences of victimization would lead to problematic online behavior. To prevent adolescents from displaying cyberbullying or being addicted to the Internet after being victimized, timely interventions aimed at relieving their psychosocial distress is very important. In particular, we should pay more attention to the depression of female victims and anxiety of male victims. Moreover, their signs of problematic online behavior should be found in a timely manner and treated. Finally, programs to promote the psychosocial competencies and life skills of young people via the positive youth development approach may help to protect young people experiencing victimization [81,82,83].

## Figures and Tables

**Table 1:** Means, Standard Deviations, and Reliabilities of all Study Variables.

Variables	M	SD	Reliabilities
1. Victimization	0.49	0.48	0.91
2. Depression	0.84	0.54	0.79
3. Anxiety	0.99	0.62	0.84
4. Internet addiction	2.04	0.77	0.90
5. Cyberbullying	1.11	0.28	0.80

**Table 2:** Correlations among all latent constructs in this study.

Variables	1	2	3	4	5
1. Victimization	-				
2. Depression	0.44 ***	-			
3. Anxiety	0.39 ***	0.75 ***	-		
4. Internet addiction	0.37 ***	0.49 ***	0.43 ***	-	
5. Cyberbullying	0.27 ***	0.22 ***	0.18 ***	0.41 ***	-

*Note.* *** *p* < 0.001.

**Table 3:** Multi-Group Test based on Gender.

Paths.	Wald *χ*^2^/*df*
Victimization→Depression (male/female: 0.44 ***/0.46 ***)	7.16 **
Depression→Internet Addiction (male/female: 0.25 ***/0.45 ***)	4.50 *
Depression→Cyberbullying (male/female: 0.10 */0.19 **)	0.03
Victimization→Anxiety (male/female: 0.39 ***/0.40 ***)	2.57
Anxiety→Internet Addiction (male/female: 0.18 **/0.03 ^ns^)	5.87 *
Victimization→Internet Addiction (male/female: 0.17 ***/0.17 ***)	0.06
Victimization→Cyberbullying (male/female: 0.20 ***/0.23 ***)	2.89
***Mediation effects***	Wald *χ*^2^/*df*
Victimization→Depression→Internet Addiction (male/female: 0.11 ***/0.21 ***)	8.69 **
Victimization→Depression→Cyberbullying (male/female: 0.05 */0.09 **)	0.06
Victimization→Anxiety→Internet Addiction (male/female: 0.07 ***/0.01 ^ns^)	4.40 *

*Note:* *** *p* < 0.001; ** *p* < 0.01; * *p* < 0.05; ^ns^ = non-significant.

## Data Availability

The data presented in this study are available on request from the corresponding author.
